# Organisational targets of patient safety improvement programs in primary care; an international web-based survey

**DOI:** 10.1186/1471-2296-14-145

**Published:** 2013-10-01

**Authors:** Joost Johan Godert Wammes, Wim Verstappen, Sander Gaal, Michel Wensing

**Affiliations:** 1 Scientific Institute for Quality of Healthcare, Radboud University Nijmegen Medical Centre,P.O. Box 9101, 114 IQ Healthcare, 6500, HB Nijmegen, The Netherlands; 2 Department of Primary and Community Care, Radboud University Nijmegen Medical Centre, P.O. Box 9101, 117 Department of Primary and Community Care, 6500, HB Nijmegen, The Netherlands

**Keywords:** Patient safety, Primary care, Audit, Feedback, Prospective risk analysis

## Abstract

**Background:**

Organisational problems contribute to many errors in healthcare delivery. Our objective was to identify the most important organisational items in primary care which could be targeted by programs to improve patient safety.

**Methods:**

A web-based survey was undertaken in an international panel of 65 experts on patient safety from 20 countries. They were asked to rate 52 patient safety items on a five-point Likert scale which regards importance of each item for use for educational interventions to improve patient safety.

**Results:**

The following 7 organizational items were regarded ‘extremely important’ by more than 50% of the experts: the use of sterile equipment with small surgical procedures (63%), the availability of adequate emergency drugs in stock (60%), regular cleaning of facilities (59%), the use of sterile surgical gloves when recommended (57%), the availability of at least one adequately trained staff member to deal with collapse and need for resuscitation (56%), adequate information handover when a patient is discharged from the hospital (56%) and periodically training of GPs in basic life support and other medical emergencies (53%).

**Conclusion:**

Seven organisational items were consistently prioritized; other items may be relevant in specific countries only. The logical next step is to develop and evaluate interventions targeted at these items.

## Background

Patient safety receives increased attention worldwide, also in primary care [1,2]. Patient safety in primary care is crucially important, as most diseases are managed in this setting, particularly in countries with a strong primary care system [3]. Primary care has low risk of major harm in most contacts and procedure, but incidents with major consequences do occur from time to time [4-6]. So, the challenge is to enhance patient safety in the high-volume, low-risk setting of primary care, while avoiding defensive medicine or heavy bureaucratic control structures.

Interventions to improve patient safety include reporting and analysis of incidents, and measurement and feedback on patient safety culture [1]. In primary care in the Netherlands, incident reporting is new and not yet widely implemented, although it is now promoted as a team-based approach to enhance patient safety through reflective learning [7]. Some research evidence supports the belief that incident reporting improves patient safety [8]. Apart from incident reporting, organisational culture has received much attention. There is also some evidence that culture within organizations may be a relevant factor in health care performance, yet articulating the nature of that relationship proves to be difficult and interventions addressing safety culture are not clearly effective [9].

Organizational problems, such as suboptimal control of repeat prescriptions or inadequate routines for cleaning equipment, contribute to patient safety incidents. The challenge is to identify methods to identify and remedy such organizational problems, which are effective as well as feasible in primary care. An inventory in an international panel of primary care experts found that continuing education of health professionals on patient safety was perceived as highly relevant and currently lacking [10]. Focusing educational interventions (e.g. audit and feedback, prospective risk analysis) on organisation of primary care is potentially feasible and effective to improving patient safety. The question is which items need to be considered in educational programs to enhance patient safety.

In the context of Linneaus Euro-PC, an international patient safety promoting and researching initiative on patient safety in primary care, we asked respondents to rate organisational items [11]. Our aim was to document the relevance of specific organisational items for patient safety to support the development of educational programs for enhancing patient safety in primary care.

## Methods

### Study design and setting

A web-based survey was conducted in a purposeful sample of primary care physicians and researchers with a special interest in patient safety. With help of the Linneaus project we identified the participants. Key individuals (all members of the Linneaus consortium) were asked to provide the names of at most 10 practising primary care physicians with a potential interest in patient safety and at most 10 researchers or experts in patient safety in their country. A total of 111 individuals were e-mailed and invited to complete an internet survey. Non-respondents were sent a second invitation after one week and a third invitation one month later. The Medical Ethics Committee of the Radboud University Nijmegen Medical Centre approved this study.

### Questionnaire

Specific organisational items were based on EPA safety indicators - European Practice Assessment, an internationally validated set of indicators developed to compare and improve the organisation and management of general practices - and an interview study in primary care physicians and nurses to explore what constitutes ‘patient safety’ in primary care [12,13]. The semi-structured interviews yielded a wide range of items considered relevant for patient safety. Salient organizational aspects were selected from these studies and translated into 52 specific items, which was subsequently reviewed by three experts on patient safety in order to fine-tune the questionnaire. These organisational items were clustered into ten broad domains: hygiene, information technology, emergency medicine, incident reporting, patient records, coordination, decision support tools, quality improvement, medication and accessibility/ triage. The respondents were asked to rate these items with respect to importance for patient safety along a five-point Likert scale which ranged from ‘not important’ to ‘extremely important’. The survey also included some questions to determine the demographic characteristics of the respondents. The following text was provided with the questionnaire: ‘We use a broad definition of patient safety. A patient safety incident is defined as an unintended event during the care process that resulted, could have resulted, or still might result in harm to the patient. Both acts of omission and of commission are included’.

### Data analysis

The data were entered into SPSS 16.0 for analysis and the response frequencies per item were calculated. As the numbers of responses per country are low, we did not compare the outcomes between countries.

## Results

The survey was completed by 65 individuals (59% response rate). Table 1 reports on their characteristics. Most (n = 48) had medical training, of which 41 were practicing general practitioners (GPs) or family/ primary care physicians. Seven had a biomedical, behavioural, social science, or information technology backgrounds and five had a background in pharmacy. The remaining three respondents had a background in allied health professions. Those working in a practice worked in practices that were spread across rural areas, towns and cities. There was a wide spread in the number of patients per practice. Table 2 reports on the reported perceived relevance of patient safety items. Table 3 reports on the average score per pre-defined domain. We will discuss the most salient findings below. We will discuss the domains in order to average rating.

**Table 1:** Characteristics of the participants of the web-based survey (N = 65)

**Gender**	
Male	31 (47.7%)
Female	34 (52.3%)
Mean age (years)	48.05 years ( ± 9.64)
Professional discipline	
Medicine	48 (76%)
Allied health profession	3 (5%)
Biomedical, behavioural or social science and eHealth	7 (12%)
Pharmacy	5 (8%)
Current profession^1^	
General practitioner/ family or primary care physician	41 (63%)
Medical teacher	17 (26%)
Employed (or director) at organization of health profession	10 (16%)
Policy advisor, policy researcher and/or public health expert	7 (11%)
Scientific researcher	20 (31%)
Nurse working in primary care, auditor and forensic doctor,	2 (3%)
(Community) pharmacist, dentist	5 (8%)
Patient	2 (3%)
For those working in a general practice, the practice size	
Mean number of registered patients (N = 34)	6399 ( ± 17060)
Mean number of patients attending every three months (N = 29)	2395 ( ± 5440)
For those working in a general practice, the location of the practice	
City/ highly urbanized	25 (56.8%)
Town (up to 10.000 inhabitants)	11 (25%)
Village/ rural area	8 (18.2%)
Country	
Germany	11 (17%)
The Netherlands	10 (15%)
United Kingdom	6 (9%)
Bulgaria	4 (6%)
Austria	4 (6%)
Latvia	3 (5%)
Spain	3 (5%)
Denmark	2 (3%)
Slovenia	2 (3%)
Slovakia	2 (3%)
Albania	1 (2%)
Belgium	1 (2%)
France	1 (2%)
Ireland	1 (2%)
Italy	1 (2%)
Luxemburg	1 (2%)
New Zealand	1 (2%)
Poland	1 (2%)
Romania	1 (2%)
Russia	1 (2%)
Unknown	8 (12%)

^1^Respondents were allowed to choose all the options that apply.

**Table 2:** Results of the web-based survey in the Linneaus project (n = 65)

	**Item**^**1**^	**Number**	**Percentage scored**
			**“Extremely important”**
1	The practice uses only sterile equipment with small surgical procedures. (A )	41/65	63.1%
2	The practice has adequate emergency drugs in stock. (C)	39/65	60.0%
3	Facilities are regularly cleaned (e.g. spirometry). (A)	38/65	58.5%
4	Sterile surgical gloves are used when recommended in prevailing guidelines. (A)	37/65	56.9%
5	There is at least one adequately trained staff member available to deal with collapse and need for resuscitation. (C)	36/64	56.3%
6	When a patient is discharged from the hospital adequate information is handed over to the GP. (F)	35/63	55.6%
7	GPs are periodically trained in basic life support and other medical emergencies. (C)	34/64	53.1%
8	Major diseases and health problems are labeled in the patient record (for example in a problem list). (E)	28/65	43.1%
9	A hygiene protocol is known to all clinicians at the practice. (A)	28/65	43.1%
10	The patient record system has a facility for information back-up. (B)	27/63	42.9%
11	An AED is present in the practice. (C)	27/65	41.5%
12	There is a system for showing alerts to inform GPs of seriously abnormal test results. (G)	27/65	41.5%
13	The practice analyses the reported incidents, and takes adequate actions. (D)	25/63	39.7%
14	Every clinical telephonic advice given is noted in the patient record. (E)	25/65	38.5%
15	There is an actual list of medication used present in the practice for every patient. (I)	23/63	36.5%
16	The practice has a control programme for oral anticoagulants when prescribed and dosed by the practice. (I)	23/63	36.5%
17	The practice has all documentation included in an electronic patient record system. (B)	23/63	36.5%
18	All prescriptions are done in an electronic prescribing system in the practice. (B)	22/63	34.9%
19	The practice analyses patient complaints. and takes adequate actions. (D)	22/63	34.9%
20	There is an explicit procedure for supplying and checking the content of the doctor’s bag. (C)	20/64	31.3%
21	The practice has an emergency telephone line. (J)	19/65	29.2%
22	Is it easy to get in contact with out-of-hours service. (J)	19/65	29.2%
23	Reminders and alerts regarding safety issues are integrated in the patient record system in the practice. (G)	18/65	27.7%
24	Patients can report incidents or complaints at the practice. (D)	17/63	27.0%
25	Computerized decision support regarding medication safety is present in the practice. (G)	17/65	26.2%
26	The practice has a control program for diabetes patients with regular HbA1c levels. (I)	14/63	22.2%
27	There is a adequate triage on the telephone to assess the urgency of the complaints. (J)	14/65	21.5%
28	The practice uses a national or international classification of diseases in the patient records. (E)	14/65	21.5%
29	The practice uses a procedure for reviewing repeat prescribing. (I)	13/63	20.6%
30	The practice has an incident register and healthcare workers reports incidents. (D)	13/63	20.6%
31	Out-of-hour care providers have access to the patient record. (E)	13/65	20.0%
32	The practice performs a periodic review of medication with pharmacists in patients who use risk full (combinations of) medication. (I)	12/63	19.0%
33	Clinical guidelines on the most prevailing diseases are present in the practice. (G)	12/65	18.5%
34	Patient complaints are registered in the practice. (D)	11/63	17.5%
35	The practice has a system for annual control of potassium and kidney function in patients using diuretics. (I)	11/63	17.5%
36	Measurement and feedback on safety culture in practice is done. (H)	11/64	17.2%
37	There is a system for recalling patients who need blood test monitoring. (G)	11/65	16.9%
38	If more than one health professional is involved in the treatment, one is clearly the central care provider. (F)	10/63	15.9%
39	Written protocols are present for the most high risk processes in care delivery. (H)	10/64	15.6%
40	There is a possibility to do diagnostic tests immediately if necessary (e.g. for C-Reactive Protein and D-dimer). (C)	10/64	15.6%
41	Known prevalence of major chronic diseases. like diabetes and depression. are documented in the practice and in line with national figures. (G)	9/65	13.8%
42	In the practice data are collected and analyzed regarding patient safety: for example: deceased patients, unplanned hospital admissions, delayed or missed diagnosis. (H)	8/64	12.5%
43	There is a system for recording outgoing requests and incoming results for diagnostic tests. (G)	8/65	12.3%
44	Patients in the practice are actively invited to raise concerns regarding patient safety as members of advisory groups. (D)	7/63	11.1%
45	The practice has a system to avoid that NSAID is prescribed without gastric protection when recommended. (I)	6/63	9.5%
46	The practice has an electronic prescribing system that is directly linked to a pharmacy. (I)	6/63	9.5%
47	The practice has working agreements with pharmacists. (I)	6/63	9.5%
48	The GP improves his knowledge on rare diseases when such a disease is diagnosed in one of the patients. (H)	6/64	9.4%
49	Patients with 3 or more different professional care providers are periodically discussed in a team meeting. (F)	5/63	7.9%
50	Every contact on the phone (for example with the practice nurse) is authorized by a GP on the same day. (J)	4/65	6.2%
51	A translator service is available in the practice. (J)	2/65	3.1%
52	The practice undergoes periodic audits by an external inspection authority. (H)	1/64	1.6%

The items are ranked by importance score.

^1^ Domain A: hygiene, B: Information technology, C: emergency medicine, D: Incident reporting, E: patient records, F: coordination, G: decision support tools, H: quality improvement, I: medication, J: Accessibility/ triage.

**Table 3:** Average score per pre-defined domain

	**N**	**Number of items**	**Theoretical range**	**Mean**	**Std. deviation**
A Hygiene	65	4	0-4	3.42	0.70
B Information technology	63	3	0-4	3.18	0.69
C Emergency medicine	65	6	0-4	3.16	0.60
D Incident reporting	63	6	0-4	3.02	0.57
E Patient records	65	4	0-4	2.92	0.65
F Coordination of care	63	3	0-4	2.88	0.64
G Decision support tools	65	7	0-4	2.87	0.54
H Quality management	64	5	0-4	2.68	0.49
I Medication	63	9	0-4	2.68	0.66
J Accessibility / triage	65	5	0-4	2.49	0.59

0 corresponds to ‘Not important’, 4 to ‘Extremely important’. The domains are sorted to score.

### Domain A: Hygiene (4 items)

The use of sterile equipment with small surgical procedures was scored as the most relevant item. All hygiene items were scored among the 10 most important items.

### Domain B: Information technology (3 items)

The item ‘The patient record system has a facility for information back-up’ scored highest in this group and tenth overall. Many respondents scored ‘The practice has all documentation included in an electronic patient record system’ and ‘All prescriptions are done in an electronic prescribing system in the practice’ as extremely important for patient safety.

### Domain C: Emergency medicine (6 items)

The item ‘availability of adequate emergency drugs in stock’ was scored highest in this group and second overall. Next to this, the items ‘There is at least one adequately trained staff member available to deal with collapse and need for resuscitation’ and ‘GPs are periodically trained in basic life support and other medical emergencies’ were scored extremely important.

### Domain D: Incident reporting (6 items)

The item ‘the practice analyses the reported incidents, and takes adequate actions’ was scored highest in this group and thirteenth overall.

### Domain E: Patient records (4 items)

The item ‘Major diseases and health problems are labelled in the patient record (for example in a problem list)’ was scored highest in this group and eighth overall. Next to this, the item ‘Every clinical telephonic advice given is noted in the patient record’ was scored relatively high.

### Domain F: Coordination of care (3 items)

The item ‘When a patient is discharged from the hospital adequate information is handed over to the GP’ was scored highest in this group and sixth overall.

### Domain G: Decision support tools (7 items)

The item ‘There is a system for showing alerts to inform GPs of seriously abnormal test results’ was scored highest in this group and twelfth overall.

### Domain H: Quality management (5 items)

All items were scored extremely important by less than 20% of the respondents.

### Domain I: Medication (9 items)

The items ‘There is an actual list of medication used present in the practice for every patient’ and ‘The practice has a control programme for oral anticoagulants when prescribed and dosed by the practice’ were scored highest in this group and fifteenth overall.

### Domain J: Accessibility/ triage (4 items)

The items ‘The practice has an emergency telephone line’, ‘Is it easy to get in contact with out-of-hours service’ and ‘There is an adequate triage on the telephone to assess the urgency of the complaints’ were scored mediocre important.

## Discussion

Items that yielded the highest scores (>50% of the experts scored the items as ‘extremely important’) were the use of only sterile equipment with small surgical procedures, the availability of adequate emergency drugs in stock, regular cleaning of facilities (e.g. spirometry), the use of sterile surgical gloves when recommended, the availability of at least one adequately trained staff member to deal with collapse and need for resuscitation, adequate information hand over when a patient is discharged from the hospital and periodically training of GPs in basic life support and other medical emergencies. We suggest that these organizational items should be targeted by educational interventions to enhance patient safety in primary care.

A previous consultation of an international panel showed that educational approaches to improving patient safety were prioritized, such as practice guidelines, a culture that enhances learning, and continuing education. Therefore we focused on such approaches in our consultation of international experts. As far as we know, this was the first study which systematically examined a broad range of organizational items in relation to patient safety in primary care in an international context. An observational study in a convenient sample 271 general practices from 10 countries found substantial variation in presence of organizational items, which was to some extent related to practice size [14]. However, this observational study was based on secondary analysis of data, while we developed our list of organizational items from the beginning with a focus on patient safety in primary care.

Some remarkable results will be highlighted. First, it was noticed that the domain hygiene scored highest of all domains; all items scored among the ten most important items. This is interesting, because hygiene is not universally perceived as highly relevant for patient safety in primary care [15]. Next to this, the domain information technology and emergency medicine scored very high as all but one item were scored ‘extremely important’ by more than thirty percent of the respondents. In many countries the items among these domains have received a lot of attention. Nevertheless, there still seem to be practices where all recommended organizational items are not implemented [15]. To our surprise, the theme ‘medication’ scored relatively low. This may indicate that the respondents do not find medication issues important for patient safety. However, it may also indicate that respondents opt for other ways of improving knowledge about medication rather than via interventions aimed at patient safety.

### Strengths and weaknesses

Our study was based on a series of studies and involved an international panel of experts, both of which contributed to the validity of findings [12]. However, the response rate was 59% so we cannot rule out selection bias. For instance, most of the respondents were primary care physician, so managers and policy makers may be underrepresented. The use of an international panel may result in the exclusion of items, which are crucially important in a particular country but not in others (for example items related to advanced technical or organizational infrastructures). However, the items that are selected by an international panel despite differences across countries are likely to be important. It might be possible that we missed important organizational items, because it is not possible to identify and assess all possible items systematically. In our study, we focused on patient safety from the provider perspective. In further research and development, the role of the patient in patient safety systems needs be further explored. Apart from this, we could not test the questionnaire at length before applying it. The low sample size did not allow reliable comparisons between for example rural and urban general practitioners, or similar comparisons.

### Implications for future research

This study highlights those patient safety items that could be included in educational patient safety interventions in primary care. Obviously, other organisational items may be particularly relevant in specific countries but less important in other countries, because primary care is organized in different ways across the world. The logical next step is to develop, apply and evaluate interventions which address the prioritized organizational items. Audit and feedback is likely to be one component of such interventions. It is helpful to consider the work by Ivers et al. [16]. which suggests that an educational intervention has highly variable impact and that specific factors seem to attribute to higher impacts: the intervention is most effective when baseline performance is low, feedback is provided by a supervisor or senior colleague, the feedback is provided more than once, the feedback is provided both verbally and written and the feedback includes both measurable targets and an action plan.

## Conclusion

This international inventory among experts of patient safety in primary care identified organizational items, which could be targeted by educational interventions. The development and evaluation of such interventions is the logical next step.

## Competing interests

The authors declare that they have no competing interests.

## Authors’ contributions

SG, WV and MW developed the study; JW collected the data, performed the analyses, presented the results and drafted the manuscript. WV and SG contributed to the conception and design of the study and critically revised the manuscript. MW supervised the study, contributed to the conception and design of the study and critically revised the manuscript. All authors read and approved the final manuscript.

## Pre-publication history

The pre-publication history for this paper can be accessed here:

http://www.biomedcentral.com/1471-2296/14/145/prepub
